# Ultrasensitive, Real-time and Discriminative Detection of Improvised Explosives by Chemiresistive Thin-film Sensory Array of Mn^2+^ Tailored Hierarchical ZnS

**DOI:** 10.1038/srep25588

**Published:** 2016-05-10

**Authors:** Chaoyu Zhou, Zhaofeng Wu, Yanan Guo, Yushu Li, Hongyu Cao, Xuefang Zheng, Xincun Dou

**Affiliations:** 1 School of Life Science and Biotechnology, Liaoning Key Lab of Bio-organic Chemistry, Dalian University, Dalian 116622, Liaoning Province, P. R. China; 2 Laboratory of Environmental Science and Technology, Xinjiang Technical Institute of Physics & Chemistry; Key Laboratory of Functional Materials and Devices for Special Environments, Chinese Academy of Sciences, Urumqi 830011, China

## Abstract

A simple method combing Mn^2+^ doping with a hierarchical structure was developed for the improvement of thin-film sensors and efficient detection of the explosives relevant to improvised explosive devices (IEDs). ZnS hierarchical nanospheres (HNs) were prepared *via* a solution-based route and their sensing performances were manipulated by Mn^2+^ doping. The responses of the sensors based on ZnS HNs towards 8 explosives generally increase firstly and then decrease with the increase of the doped Mn^2+^ concentration, reaching the climate at 5% Mn^2+^. Furthermore, the sensory array based on ZnS HNs with different doping levels achieved the sensitive and discriminative detection of 6 analytes relevant to IEDs and 2 military explosives in less than 5 s at room temperature. Importantly, the superior sensing performances make ZnS HNs material interesting in the field of chemiresistive sensors, and this simple method could be a very promising strategy to put the sensors based on thin-films of one-dimensional (1D) nanostructures into practical IEDs detection.

Chemiresistive sensors based on 1D nanostructures, such as nanotubes and nanowires (NWs), have attracted a great deal of attention because of their exquisite sensitivity and fast response to the surrounding environment^1^,^2^,^3^. The sensing layer can be based either on thin-films of 1D nanostructures^3^,^4^, or on individual 1D nanostructures^5^,^6^, which offer advantages such as high response, miniaturization of the sensor device and low power consumption. However, they are difficult to fabricate and suffering from problems such as low reproducibility and low mechanical stability^3^. Compared to the sensors based on individual 1D nanostructures, the sensors based on thin-films of 1D nanostructures offer a higher potential for practical applications, because they can easily and reproducibly be prepared by wet chemical processes, such as drop casting or spin coating^4^,^7^. However, with these preparation approaches, the 1D nanostructures on the substrate are always randomly oriented with respect to each other, and hence, the geometrical advantage of the 1D morphology is not fully utilized^3^. Therefore, it would be very attractive to fabricate thin-film sensors composed of ordered NWs over the whole sensor substrate. Niederberger *et al*. reported the W_18_O_49_ NWs thin-films with high orientational order over a macroscopic area by the Langmuir-Blodgett technique^3^. The sensor composed of 10 layers of aligned NWs was found to exhibit outstanding sensitivity to H_2_ at room temperature. However, in order to avoid the W_18_O_49_ NWs forming severe aggregation and to obtain well-dispersed NWs, further surface modification is needed, which requires a large amount of organic solvents, such as chloroform and oleylamine. Three-dimensional (3D) nanostructures containing ordered NWs are promising candidates for building thin-film sensors due to the efficient exposure of NWs and the excellent charge transport property^8^. And for the construction of 1D structure into 3D structure, the solution-based route is attractive owing to its gentle synthesis conditions, easiness to operate, and the large production amount^9^,^10^. However, to date, no successful attempt to construct 3D hierarchical structure for chemiresistive detection of vapor phase explosives has been reported.

More recently, improvised explosive devices (IEDs) are extensively used in terrorist attacks, such as those in Pune, India (2010), Oslo, Norway (2011), Boston, USA (2013) and Abuja, Nigeria (2014), owing to the readily available, low cost and legally purchased components^11^,^12^,^13^. The explosives relevant to IEDs generally consist of an inorganic oxidizer such as potassium nitrate (PN) or potassium permanganate (PP), and a fuel such as carbon source, sulphur powder (SP), sugar or powdered metals^11^,^13^,^14^. Thus, the wide variability of the constituents presents a great challenge for the effective detection of IEDs. Furthermore, compared with the great deal of literatures describing methods for the analysis of military explosives^5^,^15^,^16^,^17^,^18^,^19^,^20^, there are comparatively few publications concerning the detection of the analytes relevant to IEDs^13^,^21^. And most of these research were performed *via* ion chromatography^22^, mass spectrometry^23^, and ion mobility spectrometry^11^,^24^, which can provide sensitivity and selectivity, but require sample preparation and long analysis time at the same time^4^,^23^. Therefore, the rapid and sensitive detection of IEDs has become the most urgent demands with the boosting of the global terrorist acts, and should draw extensive attention. ZnS, which is an important II-VI semiconductor, has versatile fundamental properties for diverse applications^25^, such as water splitting^26^, bioimaging^27^, UV detecting^28^,^29^, cathodoluminescence^30^, and light-emitting materials^31^. As one of the most interesting transition-metal dopant, Mn^2+^ was often doped into the ZnS host to manipulate the optical properties, such as light-emitting and fluorescence^32^,^33^. It is reported that the fluorescent detection of military explosives, such as TNT and DNT, could be realized by Mn^2+^ doped ZnS^34^,^35^, which is mainly attributed to the introduction of the defect energy level. Apparently, Mn^2+^ doping in ZnS could also influence the electronic properties, for example, the charge carrier density, and thus the gas sensing performance. As a result, a systematical study by employing Mn^2+^ as the doping element in 3D ZnS hierarchical structure would not only help to promote the explosive vapor sensing performance, but also help to understand the chemiresistive sensing mechanisms of ZnS.

In this paper, ZnS HNs containing ordered NWs vertically aligned on the surface were prepared *via* the solution-based route and the resulting sensing performances were manipulated by Mn^2+^ doping. Based on it, ZnS HNs with different Mn^2+^ doping concentrations were fabricated into a sensory array, achieving the rapid, sensitive and recognizable detection towards vapors of the analytes relevant to IEDs, such as urea, urea fertilizer (UF), black powder (BP), PP, SP, and PN, as well as trinitrotoluene (TNT) and 2, 4-Dinitrotoluene (DNT) at room temperature.

## Results

### Tailoring of ZnS HNs by Mn^2+^ doping

**Figure 1:**
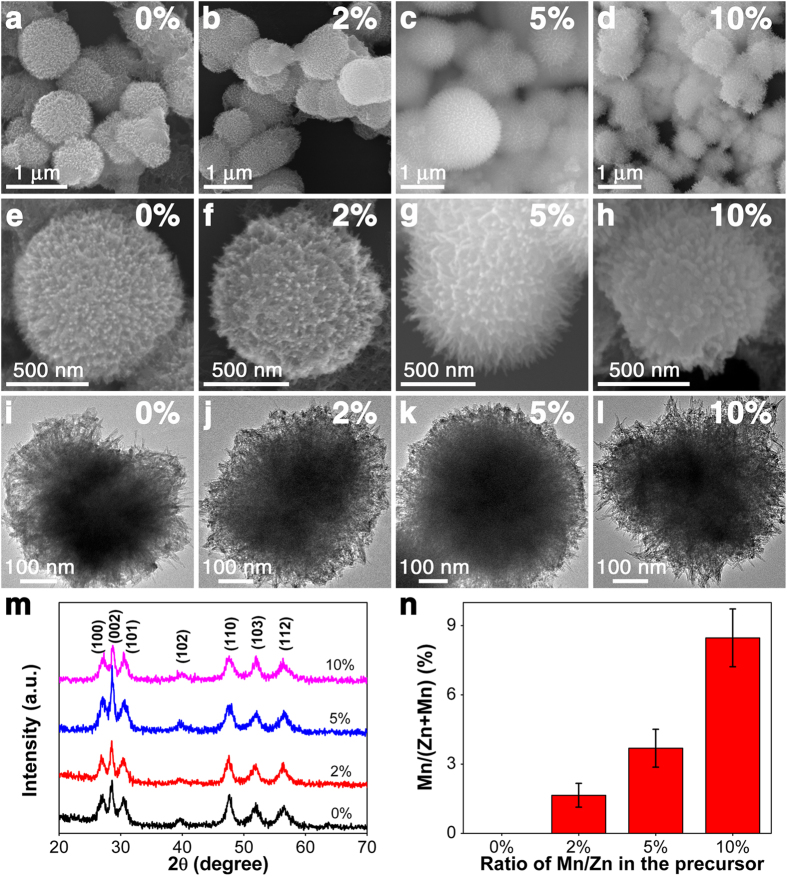
SEM and TEM images of Mn^2+^:ZnS HNs with (**a, e, i**) 0%, (**b, f, j**) 2%, (**c, g, k**) 5%, (**d, h, l**) 10% Mn^2+^, (**m**) XRD patterns, and (**n**) Content of Mn element in Mn^2+^:ZnS HNs according to the EDS analysis.

The morphology and size of the as-prepared Mn^2+^:ZnS HNs were examined by scanning electron microscope (SEM) and transmission electron microscope (TEM). The SEM images with low magnification (Fig. 1a–d) show that the final products are composed of urchin-like nanospheres with a diameter ranging from 0.5 to 1.5 μm. The enlarged SEM images (Fig. 1e–h) show clearly that the surface of the urchin-like ZnS HNs is vertically grown with ordered ZnS NWs. The TEM images (Fig. 1i–l) further confirm that the urchin-like structures are constructed by radial ZnS NWs array from the core center to the surface of HNs. The diameter of pure ZnS and Mn^2+^:ZnS NWs ranges from 4 to 6 nm (Fig. S1), which does not change obviously with the increase of the amount of doped Mn^2+^. Furthermore, the morphology and size of the as-prepared ZnS products also do not change significantly after doping with different levels of Mn^2+^.

To investigate the crystalline phases and the crystallinity of the Mn^2+^:ZnS HNs, X-ray diffraction (XRD) characterization was conducted (Fig. 1m). All the XRD peaks can be well indexed to wurtzite phases ZnS (JCPDS No. 36-1450)^10^, and no additional peaks associated with secondary phase or impurities were found, revealing the high purity of the as-synthesized samples. The elemental analysis according to the energy disperse spectroscopy (EDS) tests shown in Fig. 1n clearly demonstrates the existence of Mn in the final products,and the content of Mn element in Mn^2+^:ZnS HNs significantly increases from 0% in pure ZnS HNs to 1.65%, 3.69% and 8.47% for the 2%, 5% and 10% samples, respectively, indicating the effective doping of Mn^2+^ in ZnS HNs.

### Sensitive and real-time response towards constituents relevant to IEDs

**Figure 2:**
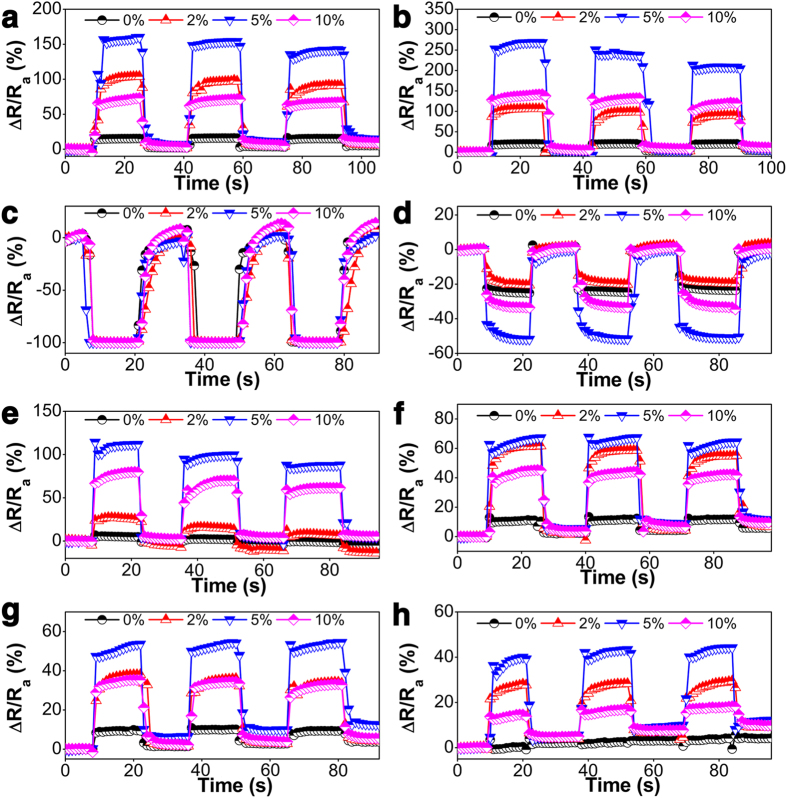
Response curves of a sensory array based on Mn^2+^:ZnS HNs during 3 successive cycles of exposure to (**a**) TNT, (**b**) DNT, (**c**) Urea, (**d**) SP, (**e**) BP, (**f**) UF, (**g**) PP, and (**h**) PN at room temperature.

To systematically investigate the gas sensing performance of the sensors based on pure ZnS and Mn^2+^:ZnS HNs, 2 military explosive vapors (TNT and DNT) and 6 improvised explosive vapors (UF, BP, PP, PN, Urea and SP) saturated at room temperature were used as the testing vapors, respectively. It can be easily observed that the responses of the Mn^2+^:ZnS HNs-based sensor are significantly enhanced compared with those of the pure ZnS HNs-based sensor towards a certain explosive vapor (Fig. 2 and Fig. S2–5). It is also observed that all of the four sensors show a good repeatability over three successive cycles. It is clearly shown that, on one hand, the responses of different sensors to the same analyte change obviously, and on the other hand, the responses of the same sensor to different analytes change remarkably. Generally, the sensing responses of all the sensors increase with the increasing of Mn^2+^ concentration in the range of 0–5% and decrease with the further increasing of Mn^2+^ concentration to 10%. When the Mn^2+^ concentration in the precursor reaches 5%, a maximum response is obtained. For example, with the increase of the doped Mn^2+^, the responses of the resulting sensors to TNT vapor achieve 12.9, 92.6, 135.7 and 64.3%, respectively, and those to BP vapor achieve 3.7, 22.7, 95.2 and 66.9%, respectively. For SP, although the response values are negative due to the relatively strong reduction, the changes of them still follow the similar trend (−24.8, −20.9, −51.3 and −34.0%, respectively) in general. This changing trend of the response values with the increase of the doped Mn^2+^ clearly indicates that Mn^2+^ concentration has an obvious influence on the sensing performance of ZnS HNs and Mn^2+^ doping is an effective strategy to manipulate the response of a sensor based on ZnS HNs.

**Figure 3:**
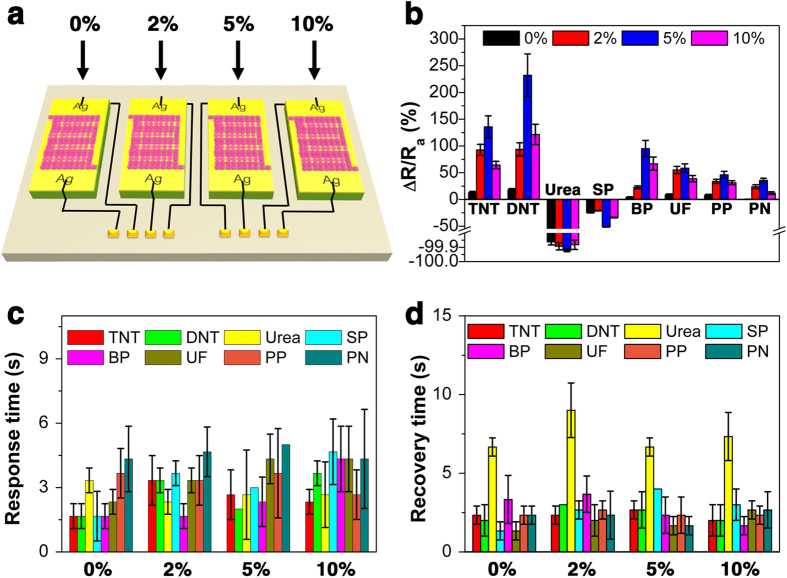
(**a**) Schematic diagram of a sensory array based on the ZnS HNs with Mn^2+^ doping content of 0%, 2%, 5% and 10%, (**b**) Response values, (**c**) Response times, and (**d**) Recovery times of the sensory array towards 8 analyte vapors at room temperature.

It is known that with the help of a sensory array, the discrimination towards different target gases could be realized. Thus, all the four sensors in the present study were organized into a sensory array (Fig. 3a). Figure 3b–d shows the statistical response values, response times and recovery times of the sensory array, respectively. One can see that the response vaules of the sensory array towards urea are limited in the range of −98% ~ −100% due to the defination of response (Fig. 3b). However, it should be noted that the resistance change towards urea is the largest, reaching the order of 3 magnitude, and this could be mainly attributed to the high hygroscopicity of urea. While the response values of the sensor based on pure ZnS HNs to TNT, DNT, SP, BP, UF, PP, and PN are just 12.9, 18.0, −24.8, 3.7, 8.6, 8.0 and 0.1%, respectively. Upon incorporation of only 3.69% Mn^2+^ (corresponding to 5% Mn^2+^ in the precursor), the response values to TNT, DNT, SP, BP, UF, PP, and PN are greatly improved to 135.7, 232.1, −51.3, 95.2, 58.5, 46.1 and 35.7%, respectively. Although the response values decrease with the further increase of doped Mn^2+^, the response values of the sensor based on the ZnS HNs with 10% Mn^2+^ to the analytes (except urea) are larger than those of the sensor based on pure ZnS HNs. This result also indicates that the doping of Mn^2+^ can effectively improve the gas sensing performance of ZnS HNs.

This sensory array also shows fast response and recovery characteristics towards all the saturated explosive vapors at room temperature (Fig. 3c,d). It is shown that the sensory array takes the longest response time (5.0 s) to detect PN and the longest recovery time (9.0 s) to detect urea at room temperature. And generally, both the response time and the recovery time of this sensory array to most of the tested constituents are less than 4 s, showing the real-time operation characteristic. Thus, it is considered that due to the efficiently exposed NWs when pasted into the sensing film and the hierarchical structure determined fast charge transport from one NW on one side of the nanosphere to that on the other side, this HN structure is beneficial to improve the sensing performance of the resulting gas sensor.

The superior sensing performances of the Mn^2+^:ZnS HNs-based sensors were further confirmed by the comparison with other recently reported chemiresistor or Schottky sensors towards explosive vapors (Table S1). It is clearly shown that the response values of the sensor based on the ZnS HNs with 5% Mn^2+^ to TNT and DNT are up to 135.7% and 232.1%, respectively. Correspondingly, the response times and recovery times to TNT and DNT are just 2.7 s, 2.7 s and 2.0 s, 2.7 s, respectively, which are remarkably superior than all of the other sensors, including the sensors based on individual 1D nanostructures^5^,^19^,^36^,^37^,^38^,^39^. This comparison further confirms that the method combing Mn^2+^ doping with 3D hierarchical structure could be a very promising strategy to put the ZnS-based explosive sensors into practical application.

### Discriminative detection of explosive species

**Figure 4:**
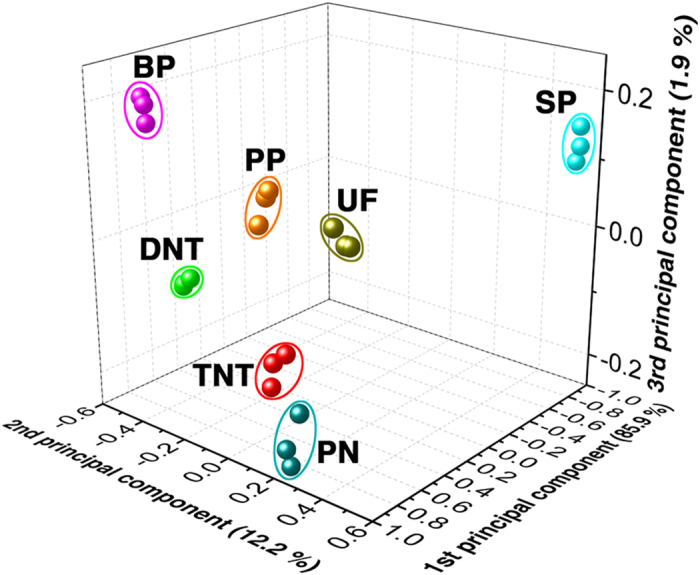
PCA analysis based on the response values of the sensory array to 7 analytes.

In order to evaluate the recognition capability of the sensory array to different analytes, the response values (except Urea) were further analyzed using a principal component analysis (PCA) method. It is shown that the clusters aggregate separately and are far from each other (Fig. 4), which indicates that the Mn^2+^:ZnS HNs-based sensory array can well recognize 7 different explosive vapors in less than 5 s. Meanwhile, it is expected that if other sensors with different doping levels are added into the sensory array, more explosives would be distinguished due to the enhanced recognizability.

### Sensing mechanisms manipulated by Mn^2+^ doping

**Figure 5:**
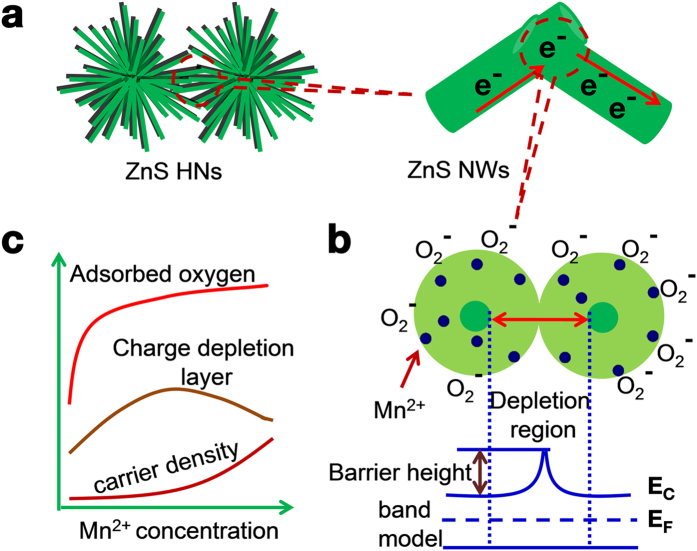
(**a**) Interfacial mechanisms for the crossed Mn^2+^:ZnS NWs, (**b**) Sensing performance manipulated by Mn^2+^ doping, (**c**) Possible effects of Mn^2+^ concentration on the adsorbed oxygen, carrier density and charge depletion layer depth.

As shown in Fig. 5a, on one hand, ZnS HNs provide well-aligned nanoporosity for effective gas diffusion, high specific surface area as well as fast electron transfer along 1D ZnS NWs, which provides the basis for the fast and high response^40^. On the other hand, when Mn^2+^ is incorporated into ZnS, there are Mn-S bonds appeared in Mn^2+^:ZnS host. Compared with that of Zn-S (2.341 Å), the bond length of Mn-S (2.431 Å) is longer^41^,^42^, thus, it is easier for the adsorbed oxygen molecules to draw electrons from Mn^2+^:ZnS NWs (Fig. 5b), which is beneficial to increase the charge depletion layer depth and obtain a high response. In addition, according to the “self-purification” effect observed in semiconductor nanocrystals^43^,^44^, the Mn impurities tend to be repelled and migrate to the surface. Therefore, when a small quantity of Mn^2+^ are doped into the ZnS host, they will migrate to the surface layer of ZnS NWs and effectively enhance the oxygen adsorption (Fig. 5c). Correspondingly, in the doping range of 0–5% Mn^2+^, the enhanced oxygen molecule adsorption dominates the sensing mechanism. In this doping range, with the increase of the amount of Mn^2+^ incorporated into the ZnS host, more oxygen molecule ions are formed on the surface of Mn^2+^:ZnS NWs, inducing a thicker electron depletion layer. As a result, the sensing performance of the sensor based on Mn^2+^:ZnS HNs becomes better with the increase of Mn^2+^and a maximum response is obtained with a Mn^2+^ amount of 5%. Meanwhile, Mn^2+^ also acts as donors in the n-type ZnS to increase the carrier density of Mn^2+^:ZnS NWs because of its weaker electronegativity. With the increase of the concentration of Mn^2+^, the carrier density of Mn^2+^:ZnS becomes larger and hence the electron depletion layer becomes thinner. Therefore, when the doping concentration of Mn^2+^ reaches up to 10%, the increased carrier density plays a more significant role in reducing the electron depletion layer, leading to the decrease of the response to the target vapors.

## Conclusion

ZnS HNs with ordered NWs vertically grown on the surface were prepared *via* the solution-based route and their chemiresistive sensing performances were manipulated by Mn^2+^ doping. Compared with the sensor based on pure ZnS HNs, the response values of the sensor based on Mn^2+^:ZnS HNs with 5% Mn^2+^ to TNT, DNT, SP, BP, UF and PP are greatly improved by about 9.5, 11.9, 1.1, 24.7, 5.8 and 4.8 times, respectively. The sensory array based on ZnS HNs with 4 doping levels of Mn^2+^ achieved the real-time, sensitive and discriminative detection of the analytes relevant to IEDs in less than 5 s. The reason for the superior sensing performance can be attributed to the efficiently exposed NWs and the hierarchical structure determined fast charge transport, as well as the Mn^2+^ doping benefited increase in charge depletion layer depth. We hope that this facile chemiresistive sensing strategy would greatly shine light on the exploration of sensing devices towards portable detection of vapors of IEDs. What’s more, it is expected that this work is going to benefit the development of thin-film gas sensors as well as the application of metal sulfide in chemiresistive sensors.

## Methods

### Materials

Zinc acetate (Zn(CH_3_COOH)_2_∙2H_2_O), L-cysteine (C_3_H_7_NO_2_S), manganese acetate (Mn(CH_3_COOH)_2_∙4H_2_O), ethanol amine, urea, potassium permanganate (PP), potassium nitrate (PN), and sulphur powder (SP) are analytical grade. 2, 4-Dinitrotoluene (DNT) was purchased from Sigma-Aldrich. Trinitrotoluene (TNT) was obtained from the National Security Department of China and recrystallized with ethanol before use. Urea fertilizer (UF) was purchased from shopping store. Black powder (BP) was self-made by blending SP, PN and graphite powder with the molar ratio of 1:2:3.

### Preparation of Mn^2+^:ZnS HNs

First, four beakers were used and 2 mmol Zn(CH_3_COOH)_2_∙2H_2_O, 4 mmol C_3_H_7_NO_2_S and a different given amount (0, 0.04, 0.1 and 0.2 mmol) of Mn(CH_3_COOH)_2_∙4H_2_O were placed in each beaker. Second, 24 mL of deionized water was added into each of the above beaker and the mixture was dispersed to form a homogeneous solution at room temperature by constant stirring for 30 min. Third, 16 mL of ethanol amine was added to the above solution and was continually stirred for 10 min. Then the resulting mixture was transferred into a Teflon-lined stainless autoclave of 60 mL capacity and maintained at 160 °C for 2 h. The system was then cooled to ambient temperature naturally. After that, the precipitation was washed with ethanol and distilled water in sequence and repeated 3 times, finally dried in oven overnight at 40 ^o^C to get pure ZnS and Mn^2+^:ZnS HNs.

### Material Characterization

X-ray diffraction (XRD) measurement was conducted using powder XRD (Bruker D8 Advance, with Cu K_α_ radiation operating at 40 kV and 40 mA, scanning from 20° to 70°). Field-emission scanning electron microscope (FESEM, ZEISS SUPRA 55VP) and transmission electron microscope (TEM, FEI Tecnai G2 F20 S-TWIN) were used to characterize the morphology of the samples. Energy disperse spectroscopy (EDS) was used to quantitatively evaluate the composition of the impurity element in the Mn^2+^:ZnS HNs. The content of Mn element in ZnS HNs was defined as the atomic ratio of Mn/(Mn+Zn) measured.

### Gas Sensor Fabrication, Test and Electric Signal Analysis

The Mn^2+^:ZnS HNs were mixed with deionized water in a weight ratio of 100:25 and ground in a mortar for 10 min to form a paste. The paste was then coated on a ceramic substrate by a thin brush to form a sensing film on which silver interdigitated electrodes with both finger-width and inter-finger spacing of about 200 μm was previously printed. The thickness of the film was controlled by the brushed cycles. The sample was dried naturally in air overnight. The sensors were aged at 4 V in air for about 24 h to ensure the good stability (Scheme S1a). The room temperature (25±3 ^o^C) saturated explosive vapor was obtained by putting solid explosive powder at the bottom of a conical flask (250 mL) and sealed for 48 h. For gas sensing test, the sensor was inserted into the saturated vapor of an explosive vapor (Scheme S1b). After the sensor resistance reached a new constant value, the sensor was then inserted into a same size conical flask full of air to recover. The electric signal of the sensor was measured by a Keithley 2636B Source Meter. The relative sensor response in resistance is defined as, Response = ΔR/R_a_ = (R_g_ − R_a_)/R_a_ × 100%, where R_a_ and R_g_ are the electrical resistances of the sensor in air and in explosive vapor. The response time is defined as the period in which the sensor resistance reaches 90% of the response value upon exposure to the explosive vapor, while the recovery time is defined as the period in which the sensor resistance changes to 10% of the response value after the explosive vapor is removed.

## Additional Information

**How to cite this article**: Zhou, C. *et al*. Ultrasensitive, Real-time and Discriminative Detection of Improvised Explosives by Chemiresistive Thin-film Sensory Array of Mn^2+^ Tailored Hierarchical ZnS. *Sci. Rep*. **6**, 25588; doi: 10.1038/srep25588 (2016).

## Supplementary Material

Supplementary Information
