# Supplementary material for: Ultrasensitive, Real-time and Discriminative Detection of Improvised Explosives by Chemiresistive Thin-film Sensory Array of Mn2+ Tailored Hierarchical ZnS
Source: Sci Rep. 2016 May 10;6:25588. doi: 10.1038/srep25588 (PMC4861922; doi:10.1038/srep25588)
Supplement: Supplementary Information [file srep25588-s1.pdf]

Supplementary Information

Ultrasensitive, Real-time and Discriminative Detection of Improvised Explosives by Chemiresistive Thin-film Sensory Array of Mn^{2+} Tailored Hierarchical ZnS

Chaoyu Zhou,[a,b‡] *Zhaofeng Wu,*[b‡] *Yanan Guo,*[b] *Yushu Li,*[b] *Hongyu Cao,*[a] *Xuefang Zheng*[a]* *and Xincun Dou*[b]*

[a] School of Life Science and Biotechnology, Liaoning Key Lab of Bio-organic Chemistry, Dalian University, Dalian 116622, Liaoning Province, P. R. China.
E-mail: dlxfzheng@126.com

[b] Laboratory of Environmental Science and Technology, Xinjiang Technical Institute of Physics & Chemistry; Key Laboratory of Functional Materials and Devices for Special Environments, Chinese Academy of Sciences, Urumqi 830011, China.
Email: xcdou@ms.xjb.ac.cn

[‡] Chaoyu Zhou and Zhaofeng Wu contribute equally to this work.

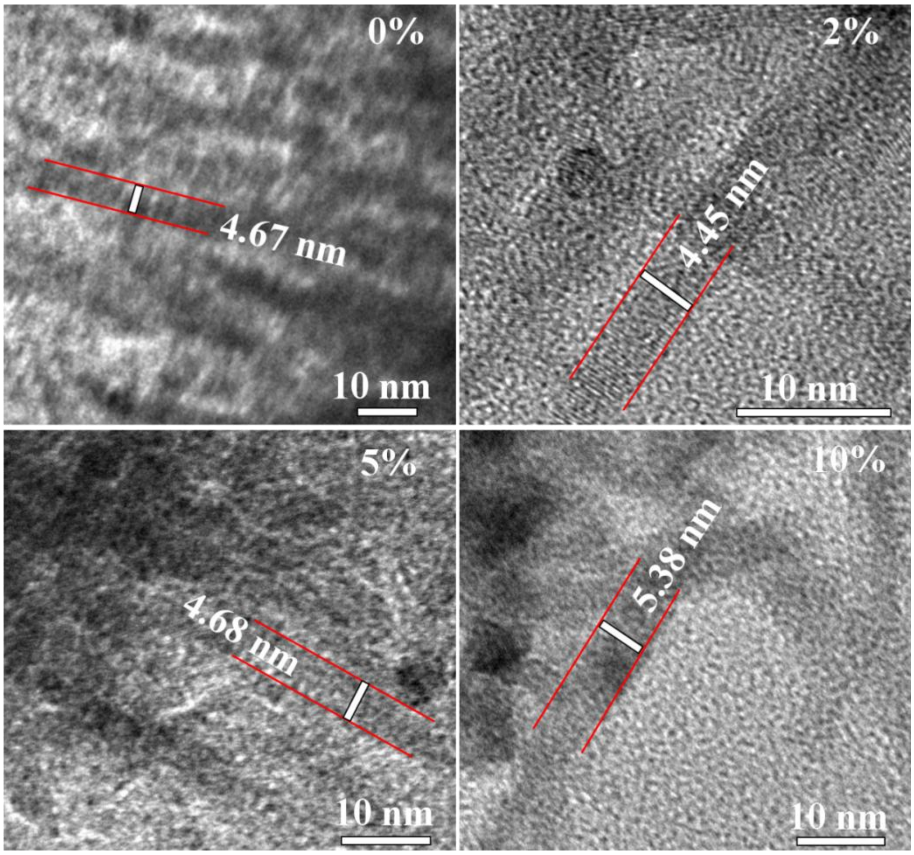

Figure S1. TEM images of NWs on the Mn^{2+}:ZnS HNs.

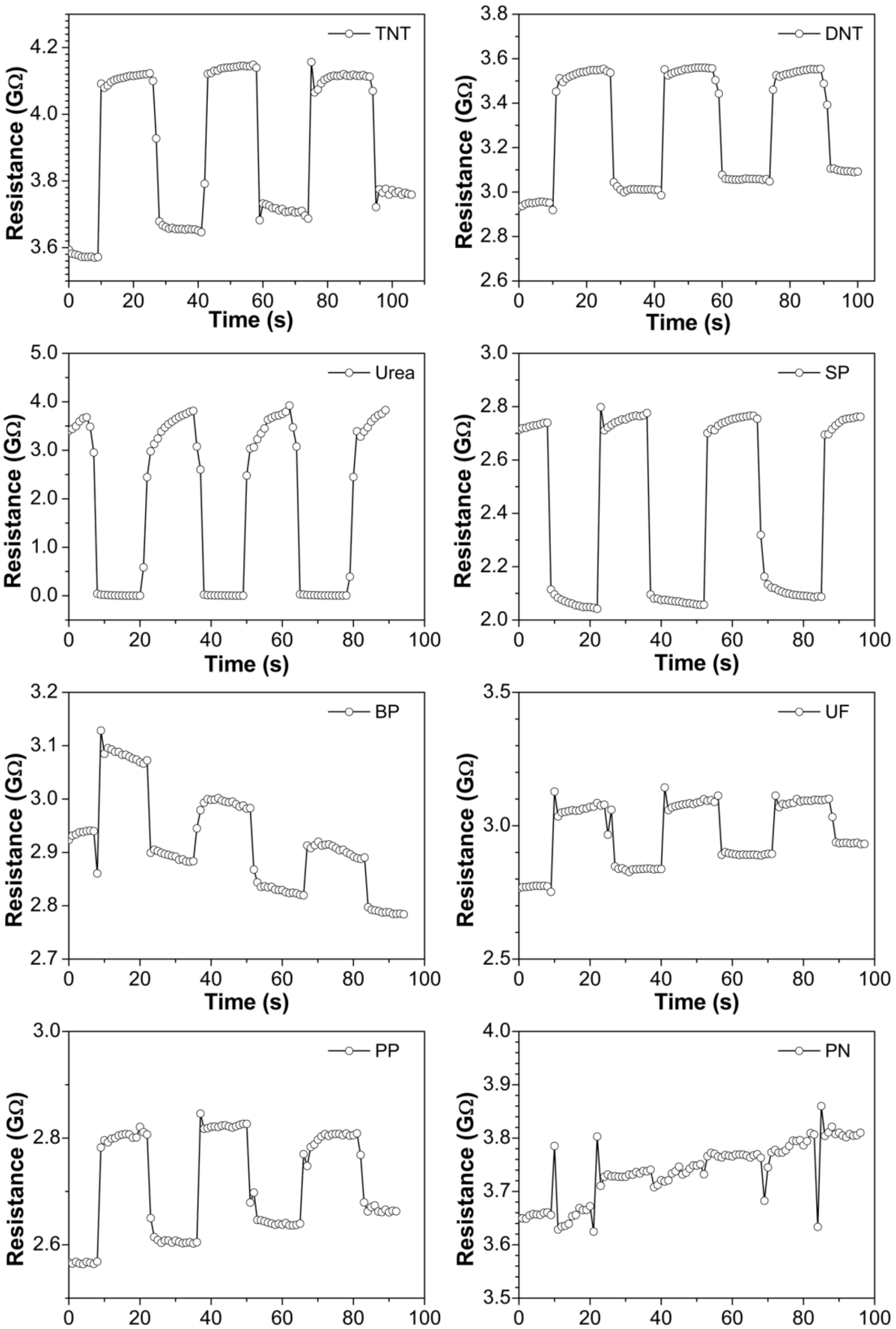

Figure S2. Response curves of the sensor based on pure ZnS HNs to TNT, DNT, Urea, SP , BP, UF, PP and PN at room temperature.

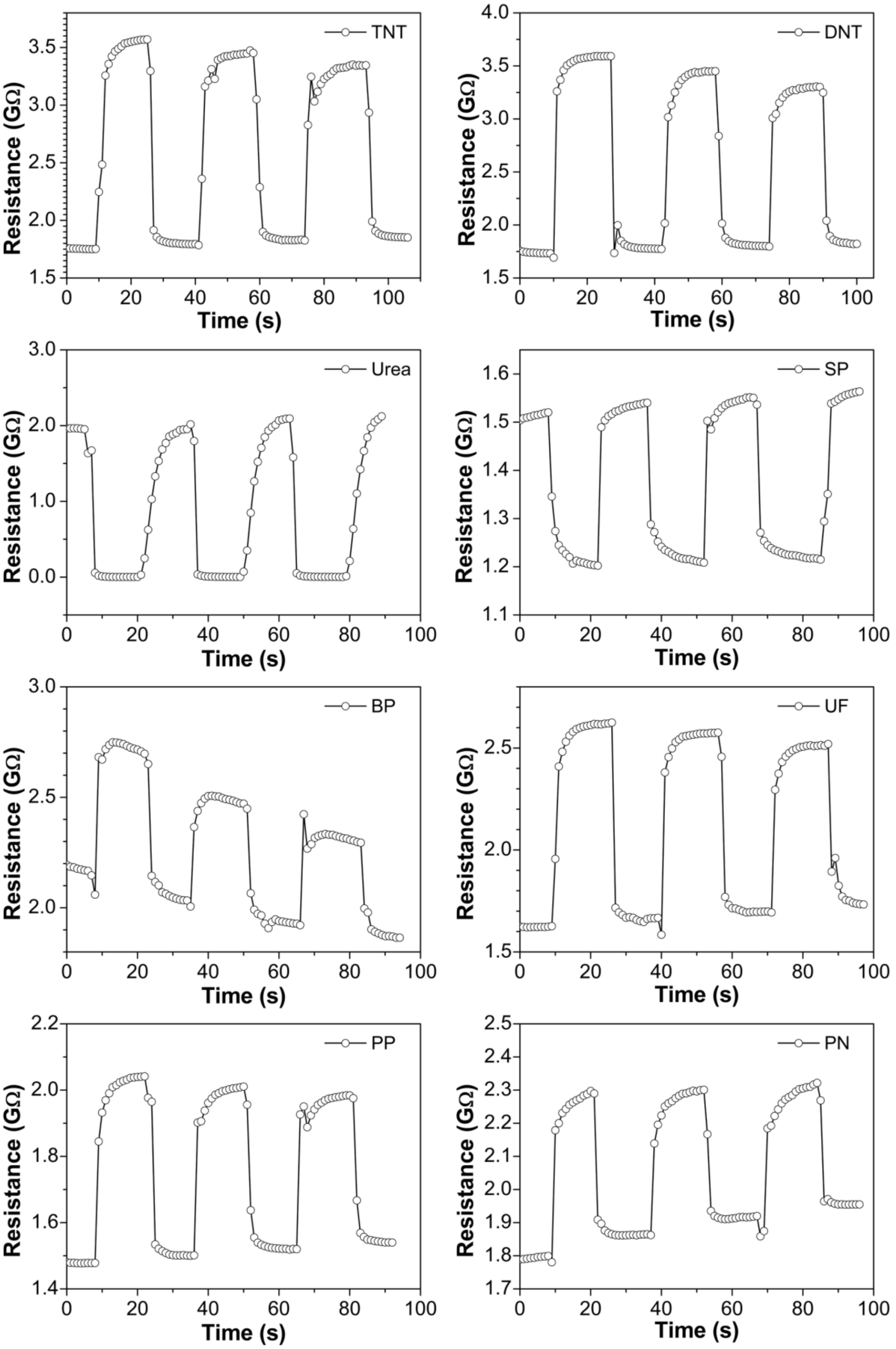

Figure S3. Response curves of the sensor based on Mn^{2+}:ZnS HNs with 2% Mn^{2+} to TNT, DNT, Urea, SP , BP, UF, PP and PN at room temperature.

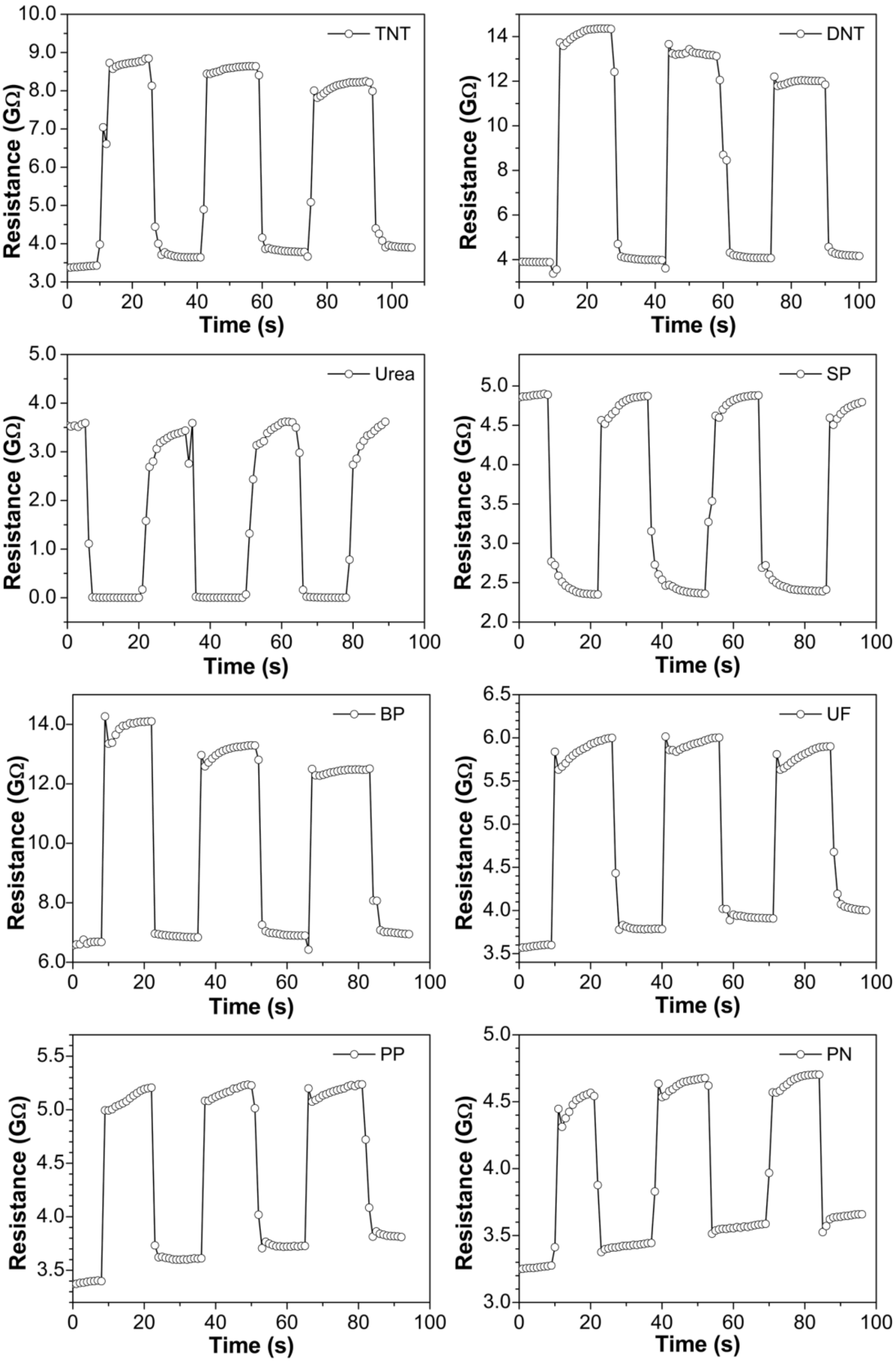

Figure S4. Response curves of the sensor based on Mn^{2+}:ZnS HNs with 5% Mn^{2+} to TNT, DNT, Urea, SP , BP, UF, PP and PN at room temperature.

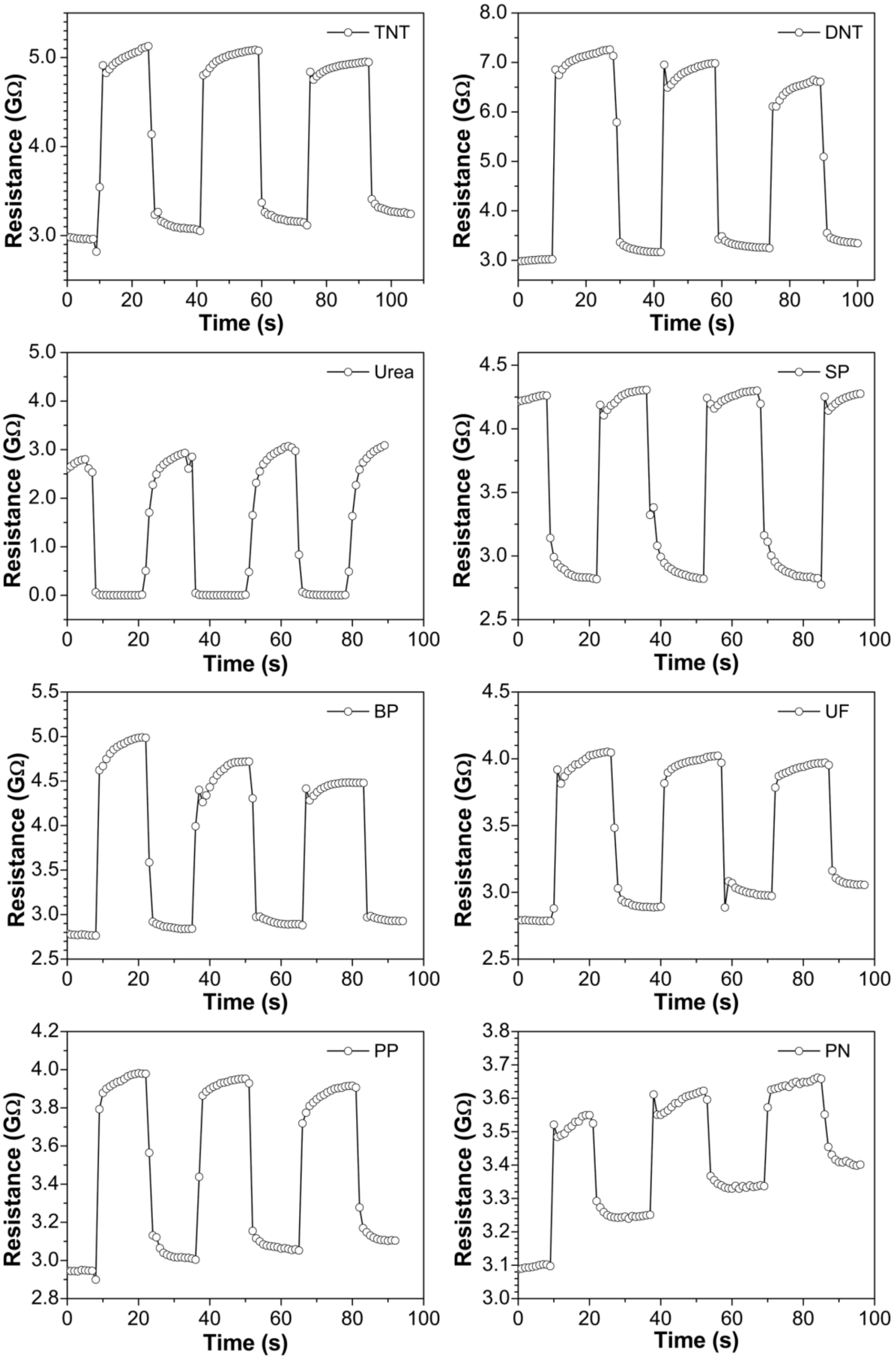

Figure S5. Response curves of the sensor based on Mn^{2+}:ZnS HNs with 10% Mn^{2+} to TNT, DNT, Urea, SP , BP, UF, PP and PN at room temperature.

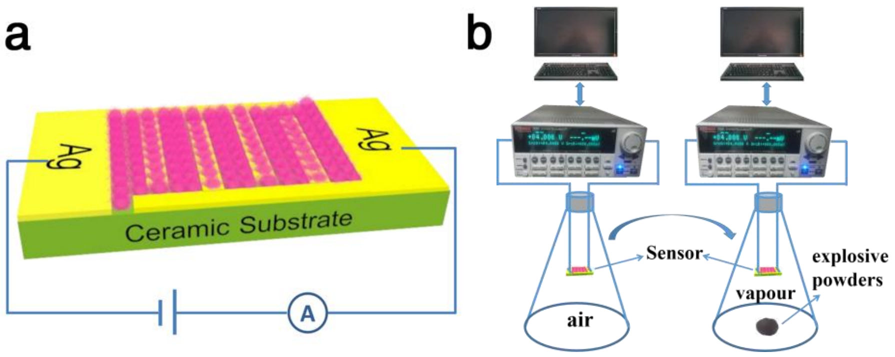

Scheme S1. Schematic diagram of (a) the sensor structure, and (b) the sensor performance measurement setup.

Table S1. Comparison of different gas-phase nitro-explosive sensors and Mn^{2+}:ZnS HNs-based sensors

Sensing materials	Analytes concentration	Response	Response time	Recovery time	Ref
ZnO nanowire	TNT, 60 ppb	20%	>10 min	-	1
SWNT	TNT, 8 ppb	8%			
Organic Nanoribbons	4-PNT, 100 ppm	15%	<5 s	>100 s	2
	DNT, 100 ppb	40%			
Titania (B) nanowires	TNT, 9 ppb	47%	~ 5 s	~ 5 s	3
	DNT, 180 ppb[a)]	38%			
SiNWs array/TiO_2/rGO	TNT, 9.1 ppb	6.3%	80 s	75 s	4
	DNT, 411 ppb[b)]	40%	120 s	80 s	
SXFA-coated microcantilevers	DNT, 300 ppt	-	35 s	35 s	5
GaN/TiO_2 NWNC hybrids	TNT, 100 ppb	10%	30 s	-	6
	DNT, 100 ppb	2%			
ZnS HNs with 5% Mn^{2+}	TNT, 9.1 ppb	135.7%	2.7 s	2.7 s	This work
	DNT, 411 ppb[b)]	232.1%	2.0 s	2.7 s	

The room-temperature saturated vapor pressure of DNT from [a)] ref.[7], and [b)] ref.[8]

1. Chen, P.-C. et al. 2,4,6-Trinitrotoluene (TNT) Chemical Sensing Based on Aligned Single-Walled Carbon Nanotubes and ZnO Nanowires. *Adv. Mater.* **22**, 1900-1904 (2010).
2. Che, Y. et al. Ultrathin n-Type Organic Nanoribbons with High Photoconductivity and Application in Optoelectronic Vapor Sensing of Explosives. *J. Am. Chem. Soc.* **132**, 5743-5750 (2010).
3. Wang, D., Chen, A., Jang, S.-H., Yip, H.-L. & Jen, A.K.Y. Sensitivity of titania(B) nanowires to nitroaromatic and nitroamino explosives at room temperature via surface hydroxyl groups. *J. Mater. Chem.* **21**, 7269-7273 (2011).
4. Yang, Z. et al. A High-Performance Nitro-Explosives Schottky Sensor Boosted by Interface Modulation. *Adv. Funct. Mater.* **25**, 4039-4048 (2015).
5. Pinnaduwage, L.A. et al. Detection of 2,4-dinitrotoluene using microcantilever sensors. *Sens. Actuators B* **99**, 223-229 (2004).
6. Aluri, G.S. et al. Nitro-Aromatic Explosive Sensing Using GaN Nanowire-Titania Nanocluster Hybrids. *Ieee Sens. J.* **13**, 1883-1888 (2013).
7. Pella, P.A. Generator for producing trace vapor concentrations of 2,4,6-trinitrotoluene, 2,4-dinitrotoluene, and ethylene-glycol dinitrate for calibrating explosives vapor detectors. *Anal. Chem.* **48**, 1632-1637 (1976).
8. Ewing, R.G., Clowers, B.H. & Atkinson, D.A. Direct Real-Time Detection of Vapors from Explosive Compounds. *Anal. Chem.* **85**, 10977-10983 (2013).
